# Fibroblast EXT1-Levels Influence Tumor Cell Proliferation and Migration in Composite Spheroids

**DOI:** 10.1371/journal.pone.0041334

**Published:** 2012-07-25

**Authors:** Cecilia Österholm, Ning Lu, Åsa Lidén, Tine V. Karlsen, Donald Gullberg, Rolf K. Reed, Marion Kusche-Gullberg

**Affiliations:** Department of Biomedicine, University of Bergen, Bergen, Norway; National Cancer Center, Japan

## Abstract

**Background:**

Stromal fibroblasts are important determinants of tumor cell behavior. They act to condition the tumor microenvironment, influence tumor growth, support tumor angiogenesis and affect tumor metastasis. Heparan sulfate proteoglycans, present both on tumor and stromal cells, interact with a large number of ligands including growth factors, their receptors, and structural components of the extracellular matrix. Being ubiquitously expressed in the tumor microenvironment heparan sulfate proteoglycans are candidates for playing central roles in tumor-stroma interactions. The objective of this work was to investigate the role of heparan sulfate expressed by stromal fibroblasts in modulating the growth of tumor cells and in controlling the interstitial fluid pressure in a 3-D model.

**Methodology/Principal Findings:**

We generated spheroids composed of fibroblasts alone, or composite spheroids, composed of fibroblasts and tumor cells. Here we show that stromal fibroblasts with a mutation in the heparan sulfate elongating enzyme *Ext1* and thus a low heparan sulfate content, formed composite fibroblast/tumor cell spheroids with a significant lower interstitial fluid pressure than corresponding wild-type fibroblast/tumor cell composite spheroids. Furthermore, immunohistochemistry of composite spheroids revealed that the cells segregated, so that after 6 days in culture, the wild-type fibroblasts formed an inner core and the tumor cells an outer layer of cells. For composite spheroids containing *Ext1*-mutated fibroblasts this segregation was less obvious, indicating impaired cell migration. Analysis of tumor cells expressing the firefly luciferase gene revealed that the changes in tumor cell migration in mutant fibroblast/tumor cell composite spheroids coincided with a lower proliferation rate.

**Conclusions/Significance:**

This is the first demonstration that stromal *Ext1*-levels modulate tumor cell proliferation and affect the interstitial fluid pressure in a 3-D spheroid model. Learning how structural changes in stromal heparan sulfate influence tumor cells is essential for our understanding how non-malignant cells of the tumor microenvironment influence tumor cell progression.

## Introduction

Cancer can be described as aberrations of normal tissue development [1], [2]. In recent years it has become increasingly recognized that the growth and malignancy of a tumor is largely dictated by the surrounding microenvironment, i.e. the tumor stroma [3], [4]. Among the major cell types that seem to be important for conditioning the microenvironment are the fibroblasts, which in tumors have been referred to by a number of names, but are now commonly called cancer-associated fibroblasts (CAFs) [5]. The normal stroma is composed of orderly structured mesenchymal cells (including adult tissue mesenchymal stem cells and fibroblasts) and extracellular substances, vascular and lymphatic networks, and minimal immune cell infiltrate. Fibroblasts are the predominant cells in the stroma and are responsible for the synthesis, deposition and remodeling of the extracellular matrix (ECM) as well as the production of growth factors, cytokines and ECM-degrading proteases [6], [7]. Cells are anchored in the ECM via specific receptors belonging to different superfamilies, including members of the integrin and heparan sulfate (HS) proteoglycan (PG) families [8], [9]. The ECM, in addition to its supportive structural role, acts as a reservoir for growth factors, guides cell migration, influences cell signaling, cell growth, cell differentiation and direct angiogenesis [8].

Heparan sulfate proteoglycans (HSPGs) are ubiquitously expressed on cell surfaces and in the ECM. They interact with a large number of ligands including growth factors, their receptors, and also structural components of the ECM. HSPGs, expressed on both tumor cells and in surrounding tissues, may thus exhibit pivotal functions in tumor growth, tumor cell adhesion and metastasis [10]. The specific roles of HSPGs within the tumor microenvironment may be manifold. Secreted HSPGs function as structural constituent of the ECM and can trap and store growth factors within the ECM. HSPGs on the cell surface of both stromal fibroblasts and tumor cells, act as co-receptors in cytokine and growth factor signaling and can thereby regulate cell proliferation, matrix production, cell migration and the invasive properties of tumor cells [10]. They may also serve as a reservoir of bound growth factors that are released by metalloproteinase-mediated matrix degradation [11].

The functions of HSPGs depend on noncovalent binding of its negatively charged polysaccharide chains with secreted proteins in the extracellular matrix and with membrane bound receptors. Protein binding to HS most likely depends on the specific structural features within the HS chains [12], [13] as well as the chain length [14]. There is a multitude of reports on alterations in HSPG expression and structure in tumor cells as compared to corresponding normal cells, which may affect the efficiency of growth factor stimulation of tumor cells [10], [11], [15], [16], [17], [18]. However, limited information is available on how structural changes in HS chains on cells in the tumor microenvironment affect tumor growth and invasiveness. Tumor progression depends on crosstalk between tumor cells and stromal fibroblasts and it has lately become evident that the stromal-derived HSPG syndecan-1 is important in signaling between stromal fibroblasts and carcinoma cells [15], [19]. Changes in sulfation pattern or chain length of stromal-derived HS will potentially lead to altered affinity for growth factors and cytokines, and consequently alter the biological activities of these factors during tumorigenesis.

The interstitial connective tissue provides the route of transport for fluids and solutes from plasma to tissues. Interstitial fluid pressure (P_if_) is important in controlling the interstitial fluid volume and is regulated through interactions between integrins on stromal cells and ECM-molecules [20]. Under normal conditions, P_if_ in skin is approximately -1 mmHg [21]. Tumor P_if_ is often elevated compared to the surrounding tissue, often as high as 20 mmHg that is commonly described as a “functional barrier” for delivery of cytostatic agents across the microvascular barrier [20], [22]. Part of this elevated P_if_ is generated by the circulatory system since the elevated P_if_ will fall by about 1/3 when the circulation is arrested [23]. However, although some information is available about the contribution from individual ECM components in generating the elevated P_if_ in tumors, much remains to be investigated. Thus, disruption of collagen fiber assembly in fibromodulin knockout mice results in a looser collagen network and lower P_if_ [24]. Furthermore, inhibition of VEGF [25], PDGF-BB [26] and TGF-β [27] results in lower and less dense ECM and a lower tumor-P_if_. In addition, cell surface HSPGs have, with contributions from integrins, been suggested to be mechanosensors for sensing interstitial flow in a 3-dimensional (3D) microenvironment [28]. However, the contribution of stromal HS chains for creating interstitial fluid pressure in different tissues is currently unclear.

The aim of the present study was to extend these studies of the role of the HSPG in tumor stroma interactions by using a 3-D spheroid system to investigate the contribution of stromal HS chains to tumor cell-fibroblast interactions and, the P_if_, in this model system.

## Results

### Cell Surface HS Expression by Tumor Cells and Fibroblasts

**Figure 1:**
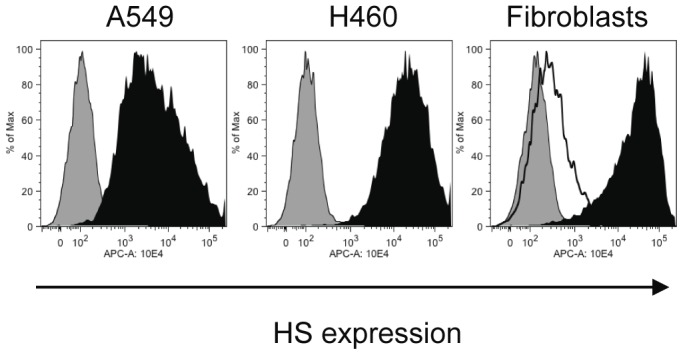
Cell surface expression of HS on wild-type fibroblasts, *Ext1^Gt/Gt^* fibroblasts, A549 and H460 tumor cells. Representative flow cytometry fluorescence histograms of 10E4 antibody binding to A549 and H460 tumor cells (black profiles), wild-type fibroblasts (black profile) and *Ext1^Gt/Gt^* fibroblasts (unfilled black curve). Controls represent cells treated only with the secondary antibody (gray profiles).

The relative amounts of cell surface HS expressed by wild-type fibroblast, *Ext1^Gt/Gt^* fibroblasts, the A549 non-small cell lung adenocarcinoma cells and the large cell lung carcinoma NCI-H460 (H460) were determined by flow cytometry using the 10E4 antibody. The 10E4 antibody, specific for HS chains, recognizes sulfated regions within HS chains [29], and is commonly used to trace HSPGs. In agreement with our previous results, wild-type (wt) fibroblasts stained strongly with 10E4 antibody whereas the *Ext1^Gt/Gt^* cells, that have very short HS chains, stained poorly with the antibody [14]. The A549 cells showed an intermediate staining indicating a cell surface HS expression in-between the two different fibroblast cell lines, whereas the HS expression of H460 cells was similar to that observed for wild-type fibroblasts (Fig. 1).

### Spheroid Formation by Tumor Cells and Fibroblasts

**Figure 2:**
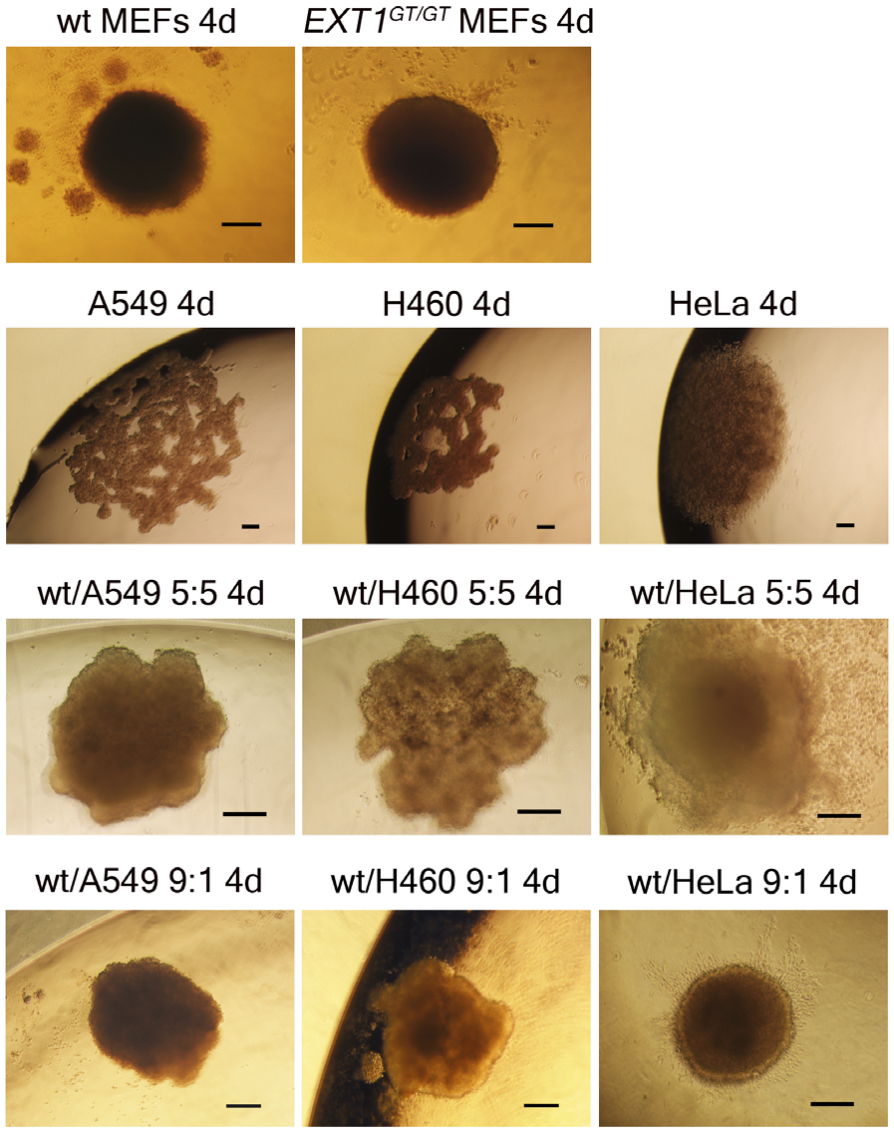
Morphology of single cell type spheroids and composite spheroids. Representative phase contrast images of multicellular spheroids generated by the hanging drop method after 4 days in culture. MEFs, mouse embryonic fibroblasts. Wild-type fibroblast spheroids, *Ext1^Gt/Gt^* spheroids and composite spheroids, magnification 10X; tumor cell (A549, H460 and HeLa) spheroids, magnification 4X: all size bars = 100 µm.

We first evaluated the ability of our genetically different fibroblasts and three human tumor cell lines, A549, H460 and the cervical adenocarcinoma HeLa, to grow as multicellular spheroids using the “hanging drop” method. Spheroid formation by the hanging drop method is a gravity driven “microtissue” formation and spheroids form homogenous spheroids of similar sizes with identical number of starting cells [30], [31]. When cells collect at the base of the hanging drop spheroid formation occur via a complex pattern of interacting cell surface molecules such as β1 integrin and/or cadherin mediated cell-cell or cell-ECM interactions [32]. Finally, compact 3D spheroids are produced by cellular contraction of the matrix [33]. Both *Ext1^Gt/Gt^* and wild-type fibroblasts spontaneously formed regularly shaped spheroids after 4 days without any significant differences in size (Fig. 2). None of the human tumor cell lines tested formed spheroids by themselves but instead formed unevenly shaped loose sheet-like cellular aggregates (Table 1, and Fig. 2).

**Table 1:** Phenotypes of tumor cell lines grown as single cell type tumor spheroids and fibroblast/tumor composite spheroid using the hanging drop method.

Cell line	Tumor type	Spheroid formation			
		100%	50%		10%
A549	Lung adenocarcinoma	No	Irregular, loose aggregates	Yes	
H460	Lung carcinoma	No	Irregular, loose aggregates	Yes	
HeLa	Cervical adenocarcinoma	No	Rounded flat, loose aggregates	Yes	

Spheroids were grown from tumor cells alone (100%), or mixed with different proportions of wild type mouse embryonic fibroblasts. 50% and 10% refer to the amount of tumor cells in the composite spheroids. Spheroid morphology was assessed and photographed under a phase contrast microscope using a Nikon D3000 digital camera 4 and 6 days after seeding.

To study the behavior of our mouse embryonic fibroblasts when in contact with tumor cells we next generated composite spheroids. To determine the fibroblast to tumor cell ratio needed to achieve tightly packed rounded spheroids we performed a series of tumor spheroid cultures with different proportions of fibroblasts. The 1∶1 ratio of fibroblasts to tumor cells enabled spheroid formation but the spheroids were loose aggregates that easily dispersed. Tighter rounded spheroids were formed with a fibroblast to tumor cell ratio of 8∶2 or 9∶1 in the seeding mixture (Table 1) indicating that fibroblasts stabilized the spheroids. Based on these results and published data demonstrating that in desmoplastic tumors fibroblasts can occupy more than 90% of the tumor mass [34], [35], the 9∶1 ratio was used in further experiments.

**Figure 3:**
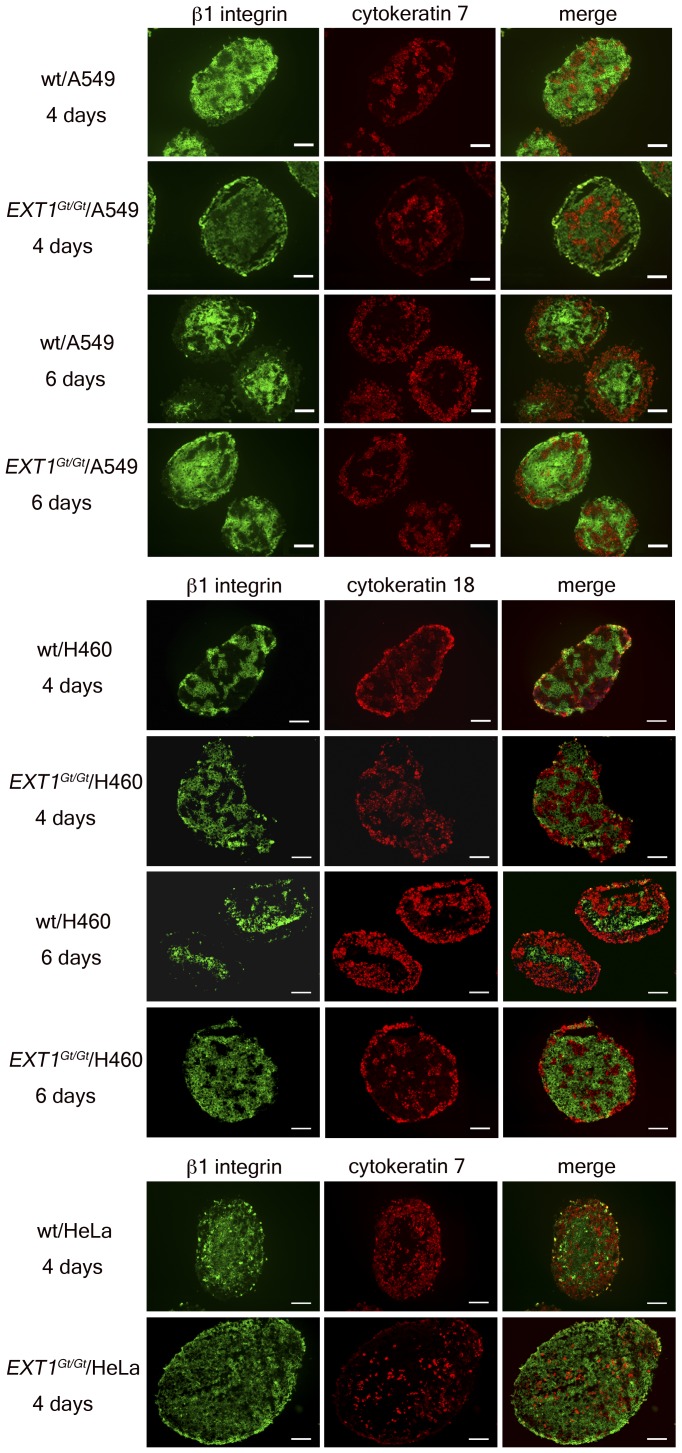
Organization of tumor cells and stromal cells in composite spheroids. Composite spheroids of mouse fibroblasts and human tumor cells (as indicated) generated by the hanging drop method, were double-stained with antibodies towards human cytokeratin 7 or 18 (red) and mouse β1 integrin (green) at day 4 and day 6. Magnification: 10X, size bars = 100 µm.Tumor cell proliferation within tumor cell/fibroblast composite spheroids.

Immunohistochemical staining of spheroid-sections using antibodies to mouse β1 integrin (specific for fibroblasts) and to human keratins (specific for epithelial cells) was used to assess the impact of the *Ext1^Gt/Gt^* mutation on tumor cell-fibroblast interactions (Fig. 3). Surprisingly, quite dramatic effects of the *Ext1^Gt/Gt^* mutation were observed in 4- and 6-days old composite spheroids. In *Ext1^Gt/Gt^*/tumor cell composite spheroids the tumor cells were scattered inside the spheroids with a tendency to migrate out to the periphery of the spheroids (Fig. 3). By contrast, for wt/tumor cell composite spheroids, the tumor cells started to migrate out to the periphery of the spheroids at day 4 and at day 6 they completely surrounded the fibroblasts (Fig. 3). Similar results as shown in Figure 3 (fibroblast to A549 cells in 9 to 1 ratio), were observed also for composite spheroids generated by mixing fibroblasts to A549 cells in 8 to 2 ratio (data not shown). Furthermore, as evident from figure 3, at both 4 and 6 days, all *Ext1^Gt/Gt^* containing spheroids appeared larger than corresponding wt-containing spheroids. This is unlikely to be due to increased fibroblast cell proliferation as the *Ext1^Gt/Gt^* cells proliferate at a slower rate as compared to wt cells and attach poorly to collagen I [14], rather suggesting that the *Ext1^Gt/Gt^* cells form looser cell-matrix contacts and/or that tumor cell proliferation is affected.

### Tumor cell proliferation within tumor cell/ fibroblast composite spheroids

**Figure 4:**
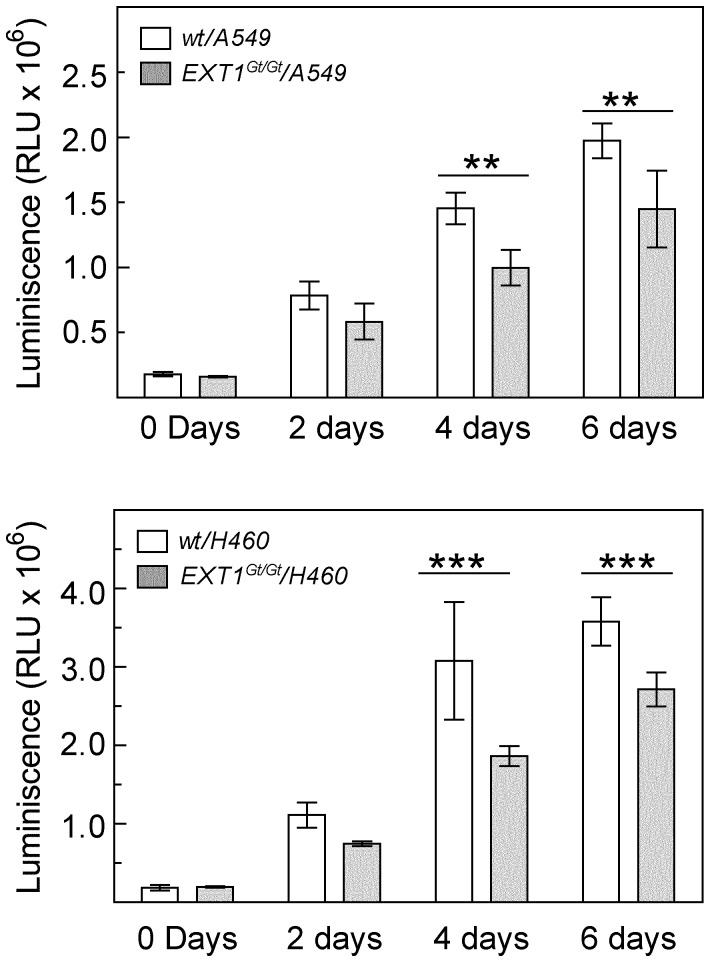
Tumor cell proliferation in composite spheroids. Composite spheroids of mouse fibroblasts and human tumor cells stably expressing the firefly luciferase gene (A549-Luc and H460-Luc) were generated by the hanging drop method and assayed for luciferase activity at the indicated time points. The figures show representative results from one out of three independent experiments. At each time point luminescence was measured from two separate spheroid preparations performed in duplicate (A549-Luc) or triplicate (H460-Luc). (**p<0.01,***p<0.001).

The staining pattern of composite spheroids indicated that there were fewer tumor cells in *Ext1^Gt/Gt^*-containing spheroids as compared to wild type fibroblast-containing composite spheroids. As the same number of tumor cells was used in the seeding mixtures we investigated if this was due to a difference in proliferation rate of the tumor cells. To assess tumor cell proliferation in composite spheroids, we used A549 and H460 expressing the firefly luciferase (A549-Luc and H460-luc, respectively). In this assay, the luciferase activity is directly related to the proliferation rate/the number of tumor cells. Our data showed that proliferation of both A549 and H460 cells were clearly attenuated in *Ext1^Gt/Gt^*/tumor cell composite spheroids as compared to wt/tumor cell spheroids (Fig. 4).

### Size and Interstitial Fluid Pressure in Spheroids Formed by Tumor Cells and Fibroblasts

**Figure 5:**
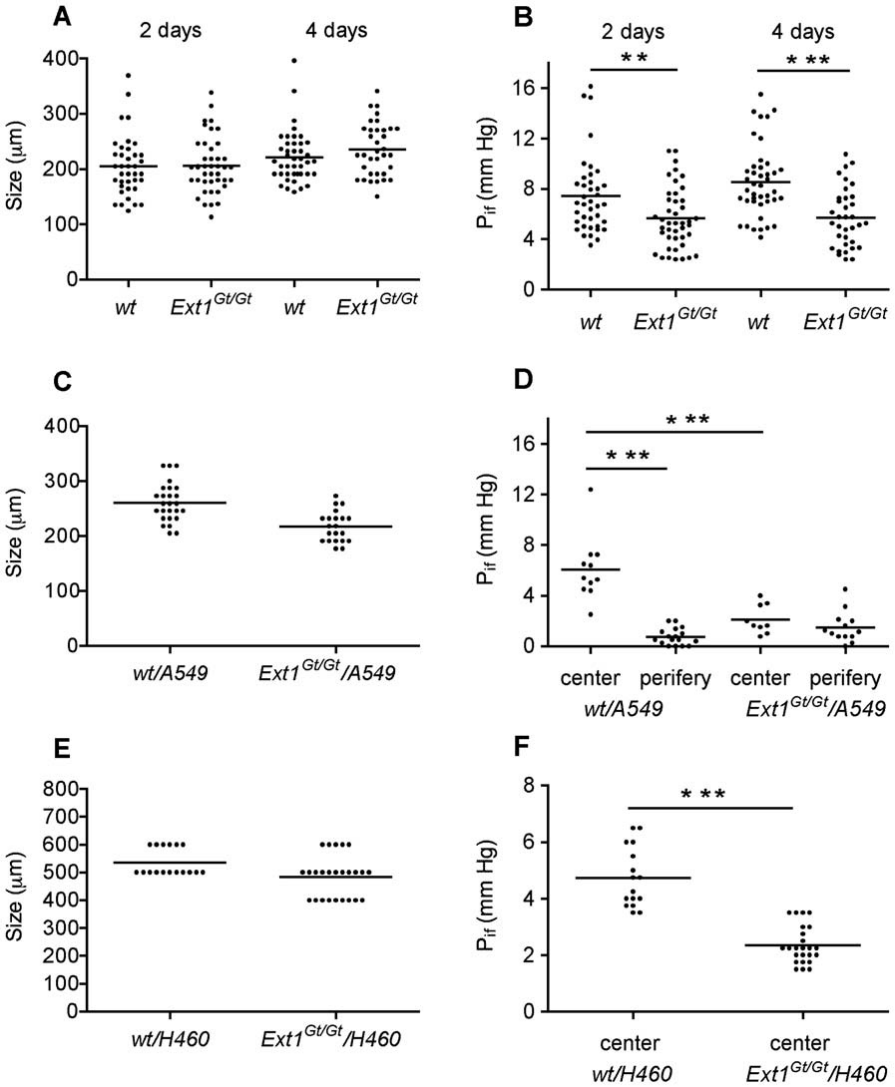
Spheroid size and interstitial fluid pressure. The size and interstitial fluid pressure (P_if_) were determined for 4-day-old spheroids generated on agar plates (A-D) or by the hanging drop method (E and F). The spheroids are as indicated. (*p<0.05, **p<0.01, ***p<0.001).

The interstitial fluid pressures (P_if_) of fibroblast spheroids seeded on soft agar plates were investigated in 2- and 4-day old spheroids. *Ext1^Gt/Gt^* fibroblasts had a bias to form larger spheroids than the wild-type fibroblast when grown on agar plates, although the changes in size were not statistically significant (Fig. 5A). It has previously been shown that larger spheroids have a tendency to have higher P_if_ than smaller spheroids [36]. Therefore, to ascertain that any changes in P_if_ between spheroids generated from wt or *Ext1^Gt/Gt^* fibroblasts were not due to size differences, similar size spheroids were used for P_if_-measurements. The *Ext1^Gt/Gt^* spheroid-P_if_ was significantly lower than that of wt spheroids both at day 2 and day 4 (Fig. 5B) further supporting the concept that *Ext1^Gt/Gt^* fibroblasts formed less densely packed spheroids.

The P_if_ in composite spheroids, *Ext1^Gt/Gt^*/A549 and wt/A549, were measured in 4-day old spheroids. In contrast to what was observed for pure fibroblast spheroids and *Ext1^Gt/Gt^*/A549 spheroids, which had a homogenous P_if_ throughout the spheroid, the P_if_ of wt/A549 spheroids varied greatly with the depth of measurement (Fig. 5D). A significant higher P_if_ was observed in the center of the wt/A549 composite spheroids than in the periphery. The average P_if_ just under the surface was 0.7 mm Hg whereas it in the center was 6.0 mm Hg. The *Ext1^Gt/Gt^*/A549 composite spheroids had an average P_if_ of 1.5 mm Hg and 2.1 mm Hg under the surface and in the center, respectively. Although there was a tendency of a lower peripheral vs. central P_if_ in *Ext1^Gt/Gt^*/A549 composite spheroids, the difference was only statistically significant for wt/A549 composite spheroids as indicated in Fig. 5D.

The *Ext1*-dependend influence on the P_if_ was not restricted to a single tumor cell line or spheroid growth condition, as similar difference in central P_if_ was observed also for H460 containing composite spheroids grown as hanging drops (Fig. 5F).

### Collagen Gel Invasion

**Figure 6:**
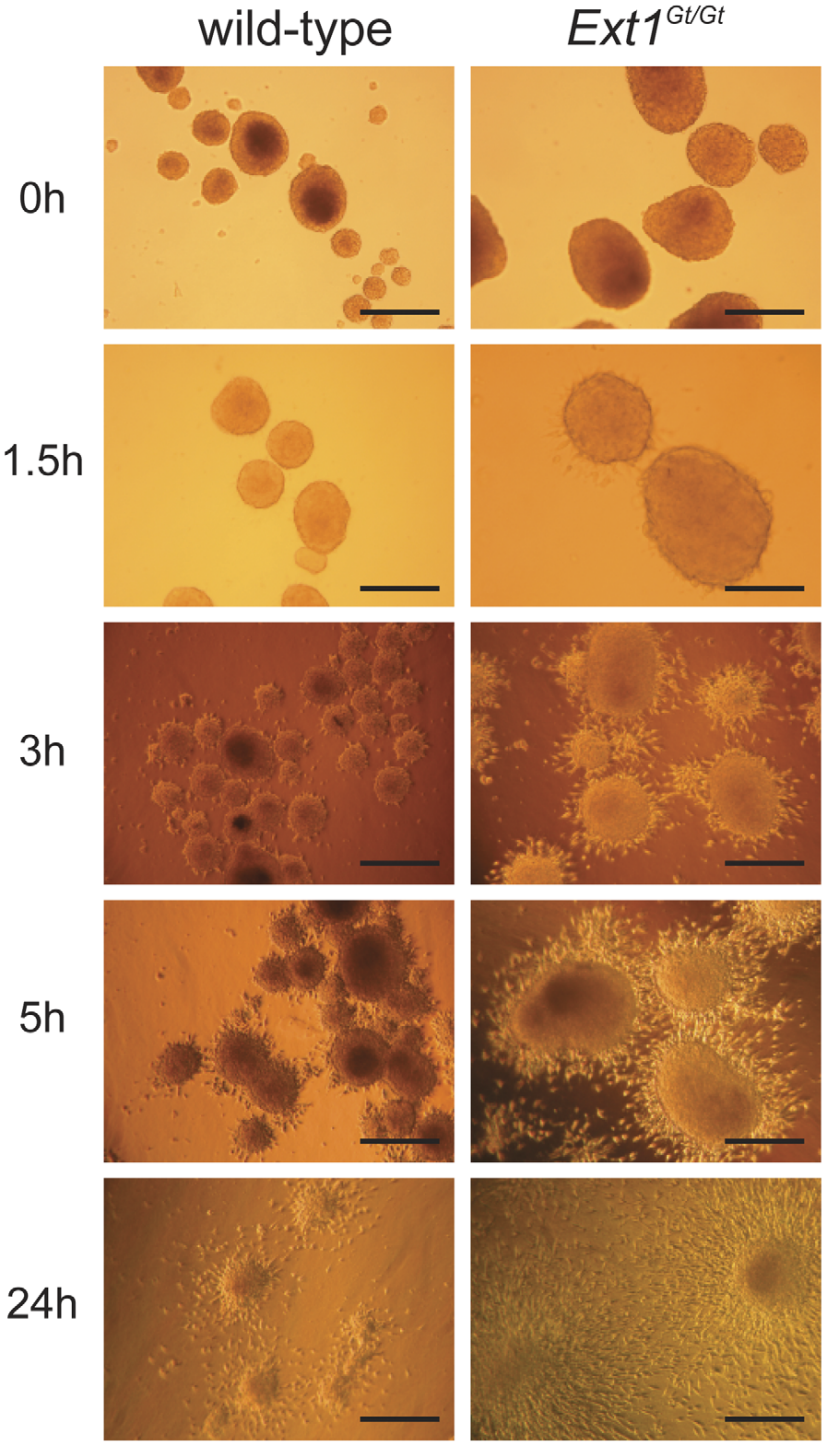
Collagen invasion by fibroblast spheroids. Four-day-old spheroids, generated on agar plates, were seeded on top of polymerized collagen type I (gels). Spheroid dissociation/fibroblast invasion into the collagen lattice was photographed under a light microscope at the indicated time points. Size bars = 200 µm.

We have previously shown that *Ext1^Gt/Gt^* fibroblasts have impaired interaction with collagen I, resulting in decreased cell adhesion and reduced ability to reorganize collagen gels [14]. We therefore examined spheroid cell invasion into collagen type I gels. Spheroids were plated on polymerized collagen I gels and the cells migrating out of the spheroids and into the collagen matrix, were monitored by phase contrast microscopy. The migration rate of the *Ext1^Gt/Gt^* cells out from the spheroid into the collagen gel was significantly higher than that of the wild-type spheroid fibroblasts (Fig. 6). The *Ext1^Gt/Gt^* cells started to migrate out from the spheroid almost immediately after seeding and 24 hours after seeding, all *Ext1^Gt/Gt^* fibroblasts had migrated into the gel, and the three-dimensional spheroid structure was not longer obvious (Fig. 6, right panel). In contrast, the outgrowth of the wild-type fibroblasts was slow and after 24 hours most of the spheroid structures were still visible (Fig. 6, left panel).

After prolonged incubation also the wild-type fibroblast completely migrated out of the spheroids and into the collagen matrix and effectively induced collagen gel contraction. One week after seeding the wild-type fibroblasts had contracted the collagen gels to approximately 10% of the initial diameter (data not shown). In contrast, the *Ext1^Gt/Gt^* cells are poor in stimulating collagen gel contraction and, in agreement with previous data, no significant contraction of collagen lattices could be detected after *Ext1^Gt/Gt^* cell invasion into collagen gels [14].

In contrast, no distinct cell migration out of the spheroids into the collagen gel was observed with *Ext1^Gt/Gt^*/A549 and wt/A549 composite spheroids. Although some cells started to migrate out of the spheroids within three hours after plating, the spheroids disintegrated and after 24 hours the collagen gels were covered with dispersed cellular aggregates (Fig. S1). One possible explanation for this effect may be the poor spheroidicity of the A549 cells alone, consistent with the previously reported dependence of tumor cells to form compact spheroids for invasion in 3-D collagen I matrices [33]. In addition, considering the fact that the tumor cells occupy the periphery of the composite spheroids, the tumor cell may act as a barrier to fibroblast migration.

## Discussion

With the aim to elucidate the role of fibroblast HS chains for stroma-tumor cell interactions and interstitial fluid pressure, we employed a 3-D spheroid tumor cell/fibroblast co-culture method, believed to mimic the initial avascular state of solid tumors. Moreover, results of previous studies suggest that, during spheroid formation, the fibroblasts undergo activation and acquire a phenotype, which resembles that of activated fibroblasts in tumor stroma [37].

We have previously reported that *Ext1^Gt/Gt^* fibroblasts exhibit short sulfated HS chains [38] and as a consequence these cells have a reduced capacity to adhere to and to remodel collagen I, although they express the collagen binding integrins α1, α2 and α11 at similar levels as the wild-type fibroblasts [14]. Immunohistochemical characterization of the composite spheroids clearly showed a difference in distribution of tumor cells and fibroblasts. The tumor cells in wt/tumor cell composite spheroids migrated out to the periphery and at day 6 completely surrounded the fibroblasts. In contrast, the tumor cells mixed with *Ext1^Gt/Gt^* cells showed a much slower rate of migration and were still intermixed with the fibroblasts also after 6 days in culture. Tumor cell proliferation was clearly attenuated in composite spheroids containing *Ext1^Gt/Gt^* cells (Fig. 4). Taken together, these observations imply that the capacity of tumor associated fibroblasts to attach to the surrounding ECM and, maybe also to each other, affects the migration and proliferate properties of tumor cells. Since the expression levels of different collagen binding integrins are unchanged in *Ext1^Gt/Gt^* cells [14], this effect might be explained by an altered activation status of the integrins and/or weakened interactions between integrins and HS co-receptors. The wt/tumor cell composite spheroids showed a clear border between the center, containing fibroblasts, and the periphery containing tumor cells, (Fig. 3) and the P_if_ differed significantly between the two compartments. The *Ext1^Gt/Gt^*-containing composite spheroids, in contrast, had a denser core of tumor cells at the center intermixed with fibroblasts and as a consequence the P_if_ did not differ between periphery and center. To the best of our knowledge, a role for HS chains in regulating P_if_ has not been described before. The P_if_ in normal tissues appears to be regulated by an integrin-mediated fibroblast interaction with the ECM in parallel with growth factor induced contractility of the cells [20], [39], [40]. In most solid tumors the P_if_ is increased and thus an obstacle to cancer chemotherapy as increased P_if_ counteracts capillary fluid filtration [20]. Thus, from a functional point of view, our data suggest that in spheroids with *Ext1^Gt/Gt^* mutated cells the ECM is less dense and has a higher hydraulic conductivity resulting in a lower pressure gradient. Our data strongly suggests that the HPSG affects the tumor stroma properties as has previously been demonstrated for collagen architecture [24] and the role of PDGF [26] and TGF-β [27]. Considering these facts, the lower interstitial fluid pressure measurements in *Ext1^Gt/Gt^* containing spheroids and the increased speed of migration out of the spheroids and into the collagen gels of *Ext1^Gt/Gt^* cells (Fig. 6) are most probably not due to an increased interaction with the collagen, but instead suggest that *Ext1^Gt/Gt^* cells display an intermediate affinity between *Ext1^Gt/Gt^* cells and collagen that is more optimal for cell migration. The molecular mechanism for the *Ext1*-dependent attachment to collagen I remains to be elucidated but, cell adhesion to ECM activate several intracellular signaling pathways that regulate cell migration through integrins and other cell surface receptors including HSPGs. The *Ext1^Gt/Gt^* cells show impaired signaling response to growth factor stimulation [14] and considering the emerging concept of the HSPG-integrin cooperation in cell signaling [16], [41], [42] the mutation may also influence the integrin-dependent cell adhesion to collagen [9], [43], [44].

The interactions between cancer cells and their microenvironment are central for tumor development [4], [45]. The proportions of different stromal cells and ECM differ widely between different tumors. For example, in desmoplastic tumors, such as many carcinomas of the breast, stomach and pancreas, the stromal compartment may account for >80% of the tumor mass, whereas in other tumors such as medullary carcinomas of the breast and many lymphomas, stroma generally represents only a minimal fraction [46]. There is no direct evidence for a close correlation between the deposition of stroma and tumor malignancy. The predominant cell type in the tumor stroma is the activated fibroblast and in the tumor stroma microenvironment there is paracrine signaling between the fibroblasts and the tumor cells. *In vitro* studies of tumor cell behavior often include 2D (monolayer) culture conditions, not taking into account that *in vivo*, tumor cells grow within a 3D matrix [47], [48]. Solid tumors *in vivo* often grow with actively proliferating tumor cells close to blood vessels. *In vivo,* the tumor cells and fibroblasts stay apart and the fibroblasts surround the tumor cells [35], [49] In addition to fibroblasts, solid tumors contain endothelial cells and immunocompetent cells that affect tumor cell growth and migration. Thus the tumor cell – fibroblast arrangement observed in our spheroid model may represent an avascular early stage of tumor development.

Enzymatic modification of tumor-derived HSPGs is an important mechanism for regulation of HSPG function in cancer. These modifications involve, release of the extracellular core protein by sheddases (matrix metalloproteinases, MMPs) [15], cleavage of cell associated HS chains by the heparanase enzyme or removal of certain sulfates groups from the HS chains by the extracellular HS-sulfatases reviewed in [10], [11], [16], [17], [18]. The molecular mechanism by which stromal *Ext1* levels, and the resulting changes in HS chain length modulate tumor cell migration and P_if_ was beyond the scope of this study but may involve, ECM assembly, growth factor signaling and/or MMPs. It is important to emphasize that the *Ext1^Gt/Gt^* mutation not only affect cell associated HSPGs, involved in cell adhesion and cell signaling, but also the secreted basement membrane HSPGs that are important modulators of cancer growth and metastasis [50]. In addition, HSPGs, in basement membranes and on cell surfaces, collaborate with other matrix components determining the structure and function of the ECM [51], [52]. MMPs are important in ECM remodeling influencing cell adhesion, migration, differentiation and tissue infiltration of tumor cells [53]. MMPs cleave ECM components and ECM-associated molecules, including cell associated HSPGs, thus liberating bioactive fragments and growth factors, all of which influence cellular behavior. HS chains also directly interact with several different MMPs which, depending on the particular MMPs and HSPGs involved, can either increase or inhibit the catalytic activity of the metalloprotease [54], [55].

In conclusion, our results agree with previous studies demonstrating a role for HSPGs for cell-ECM interactions. In addition, using fibroblasts with a mutation in *Ext1*, we herein demonstrate a central role of stromal HSPG for spheroid formation, the P_if_ and tumor cell migration within these spheroids.

## Materials and Methods

### Cell Culture

Sv40-immortalized mouse embryonic fibroblast cell lines derived from wild-type and *Ext1* gene trapped (Ext1Gt(pGT2TMpfs)064Wcs, *Ext1^Gt/Gt^*) embryonic day 11.5 mouse embryos were as described previously [38], [56]. The human non-small cell lung adenocarcinoma A549, the large cell lung carcinoma NCI-H460 (H460) and the cervical adenocarcinoma HeLa cell lines were from the American Type Culture Collection (ATCC). Monolayer cultures and multicellular spheroids were cultured in Dulbeccós modified Eaglés medium (DMEM) with Glutamax (Gibco) supplemented with 10% fetal bovine serum (FBS), 100 units/ml of penicillin and 0.1 mg/ml of streptomycin (all from PAA Laboratories).

### Preparation and Culturing of Spheroids

Single cell type multicellular spheroids from wild-type and *Ext1^Gt/Gt^* fibroblast and composite spheroids containing a mixture of fibroblasts and tumor cells were prepared using the hanging drop method [30], [31]. Single cell suspension of fibroblasts or tumor cells alone or a mixture of 10%, 50%, 80% or 90% fibroblasts combined with 90%, 50%, 20% or 10% A549 cells were diluted to 1×10^6^ cells/ml. Twenty five microliters of the cell suspension (2.5×10^4^ cells) were pipette onto the lid of a cell culture dish to form one drop. Approximately 40 hanging drops were dispensed on each lid that was then inverted and placed over a cell culture dish containing DMEM for humidity and cultured under standard conditions. H460 and HeLa cells were mixed with wild-type or *Ext1^Gt/Gt^* fibroblasts in 1/1 and 9/1 ratios of fibroblasts to tumor cells and cultured as described above.

In some experiments, as indicated in the text, single cell type and composite spheroids were prepared using the liquid overlay technique. Briefly, confluent monolayers were trypsinized, resuspended as single cells in supplemented DMEM, and 3×10^6^ cells re-plated in a drop-wise fashion to 75-cm^2^ tissue culture dishes that had been coated with 0.75% agar (Noble agar, Difco). Cells began to form spheroids after one day and the media was renewed on day two of sub culture.

### Interstitial Fluid Pressure Measurements

For interstitial fluid pressure (P_if_) measurements spheroids were transferred to 10-cm cell culture dishes and left for 2 hours in 37°C. This allowed the spheroids to attach to the plastic without starting to spread. P_if_ was measured using a micropuncture technique [36]. Briefly, sharpened glass capillaries (tip diameter 3–5 µm) filled with 0.5 M NaCl colored with Evans blue was connected to a servo-controlled counter pressure system. The P_if_ measured in the cell culture medium immediately outside the spheroid was defined as its zero pressure. The glass capillary was inserted into the spheroid with the help of a stereomicroscope (Wild M5, Heerbrugg, Switzerland). P_if_ was recorded for 30 seconds and in order for a measurement to be accepted, the following criteria had to be fulfilled: (1) feedback gain could be changed without changing the pressure; (2) applying suction to the glass capillary by the pump increased the resistance in the capillary, i.e the capillary is open; and (3) zero pressure did not change during the measurement. The size of the spheroids was measured after each P_if_ recording using an eyepiece with a grid.

### Spheroid Invasion into Collagen Gels

Collagen gels were made one day prior to seeding of the spheroids, by mixing collagen type I (PureCol, Advanced Biomatrix), Calcium- and Magnesium Free-Hanks Balanced Salt solution (CMF-HBSS), 10 X Minimum Essential Medium (MEM) (Gibco), NaHCO3, NaOH [57]. All solutions were kept on ice before and during mixing. 500 µl aliquotes of the final gel solution, containing 1 mg/ml collagen, was transferred to a 24-well plate and were left to polymerize overnight in the cell culture incubator. Spheroids were seeded on top of the collagen gels in 500 µl of culture medium and photographed with phase contrast using a Canon Powershot S50 digital camera mounted on a Leica DMIL microscope at 0, 1.5, 3, 5 and 24 hours after plating, to monitor the migration into the collagen lattices.

### Immunofluorescence

Immunofluorescence staining was performed on cryosections of 4 and 6 days old spheroids generated using the hanging drop technique. Sections with a thickness of 7 µm were fixed in acetone for 10 minutes at −20°C and were then rehydrated in PBS (3×10 min). Unspecific binding sites were blocked with 10% goat serum diluted in PBS for 1 hour at room temperature. Thereafter, the sections were incubated with primary antibodies against mouse β1 integrin (rat anti-mouse monoclonal, dilution 1∶500, from Millipore), human cytokeratin-7 (A549 and HeLa cells, rabbit anti-human polyclonal, dilution 1∶200, from Novus Biologicals) and human cytokeratin-18 (NCI-H460 cells, rabbit anti-human monoclonal, dilution 1∶200, from Epitomics) for 1 hour at 37°C. After two washing steps with PBS/0.05% Tween and an additional washing step with PBS, the secondary antibodies were applied. DyLight 488 AffiniPure Goat Anti-Rat IgG (H+L) (1∶800, Jackson ImmunoResearch,) was used as secondary antibody for β1 integrin and DyLight 549 AffiniPure Goat Anti-Rabbit IgG (H+L) (1∶800, Jackson ImmunoResearch,) was used for cytokeratin-7 and cytokeratin-18. The sections were incubated with the secondary antibody for 1 hour at RT. After two washing steps in PBS/0.05% Tween, sections were mounted in Vectashield mounting media. The stained sections were studied under a Zeiss Axioscope fluorescence microscope and micrographs were acquired using a digital AxioCam mRM camera (Zeiss).

### Flow Cytometry

For flow cytometry analyses, cells were cultured to 80% confluency in 6-well plates, harvested by trypsinization, transferred to FACS tubes, washed three times in PBS and counted. For the detection of cell surface-bound HS, 5.0×10^5^ cells were incubated for 30 min at 4°C with 1∶50 dilution of the primary antibody 10E4 (Seikagaku). After three washes with PBS, cells were incubated with 1∶75 dilution of the secondary antibody, an allophycocyanin (APC) conjugated goat-anti-mouse IgG (Jackson ImmunoResearch Laboratories, Inc.) and analyzed by flow cytometry using an AccuriC6 system (Accuri Cytometers Inc.). Data analysis was performed using FlowJo software (Tree star, Inc.). Cells incubated with the secondary antibody alone, served as negative controls.

### Proliferation Assay

To determine tumor cell proliferation in composite spheroids, A549 and H460 were stably transduced with a lentiviral expression vector (pCDH-CMV-MCS-EF1-puro (CD510B-1) HIV-based lentiviral vector, from System Biosciences expressing luciferase-EGFP fusion protein (a kind gift from Dr. Jian Wang, University of Bergen, Norway). Transduced cells (A549-Luc and H460-Luc cell lines) were isolated via EGFP expression by flow cytometry (BD FACSAria SORP, BD Biosciences, Franklin Lakes, NJ, USA). Composite spheroids (*Ext1^Gt/Gt^*/A549-Luc, wt/A549-Luc, *Ext1^Gt/Gt^*/H460-Luc and wt/H460-Luc, 9/1 ratio) were prepared by the hanging drop method as described above. Spheroids (20 drops for each preparation, 5×10^5^ cells/preparation) were collected from day 2 to day 6. Five hundred microliters of the cell suspension (5×10^5^ cells) used for preparing the spheroids was collected as the starting point for the assay (time zero). Collected spheroids were frozen at −70°C until samples for all time points were collected. Then, spheroids from each preparation were solubilized in 100 µl lysis buffer (Tropix® Lysis Solution, from Applied Biosystems). A 20 µl-portion of each cell lysate was transferred to microtiter plates to which 100 µl of luciferase substrate (Luciferase Assay System, from Promega) was added and luciferase luminescencewas measured using a Wallac 1420 VICROR3 Multilabel Counter (PerkinElmer). At each time point, two separate spheroid preparations were measured in duplicate or triplicate.

### Statistical Analysis

To identify differences between groups for (n>2), a two-way Anova with Bonferroni-Dunn correction for multiple comparisons, was used. For comparisons where n = 2, the Mann-Whitney U-test was applied. P<0.05 was considered statistical significant.

## Supporting Information

Figure S1 **Collagen invasion by composite spheroids.** Four-day-old fibroblast/A549 composite spheroids, generated on agar plates, were seeded on top of polymerized collagen type I (gels). Spheroid dissociation/fibroblast invasion into the collagen lattice was photographed under a light microscope at the indicated time points. Size bars = 200 µm.
(TIFF)
