# Supplementary figures and images for: Fibroblast EXT1-Levels Influence Tumor Cell Proliferation and Migration in Composite Spheroids
Source: PLoS One. 2012 Jul 25;7(7):e41334. doi: 10.1371/journal.pone.0041334 (PMC3405129; doi:10.1371/journal.pone.0041334)

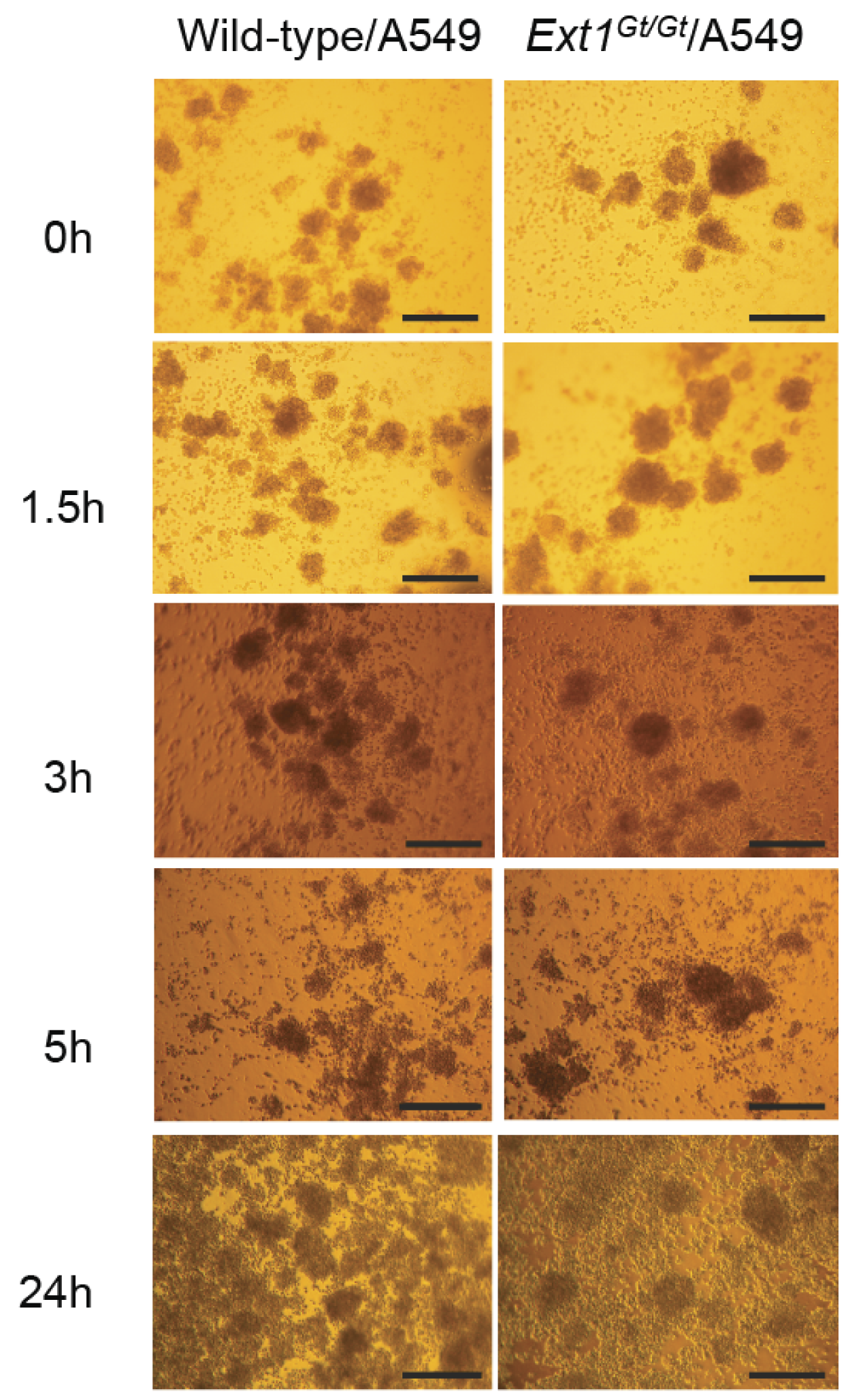

Supplement: Figure S1 — Collagen invasion by composite spheroids. Four-day-old fibroblast/A549 composite spheroids, generated on agar plates, were seeded on top of polymerized collagen type I (gels). Spheroid dissociation/fibroblast invasion into the collagen lattice was photographed under a light microscope at the indicated time points. Size bars = 200 µm. (TIFF) [file pone.0041334.s001.tiff]
